# PTSD assistance dogs: concerns for animal well-being, rights, and justice

**DOI:** 10.3389/fvets.2025.1658857

**Published:** 2025-12-17

**Authors:** Laura Kiiroja, Simon Gadbois, Andrew Fenton

**Affiliations:** 1 The Wildlife Ethology and Canine Olfaction Lab, Department of Psychology and Neuroscience, Dalhousie University, Halifax, NS, Canada; 2 Department of Philosophy, Dalhousie University, Halifax, NS, Canada

**Keywords:** animal rights, animal welfare, animal well-being, PTSD, PTSD service dogs, trauma

## Abstract

PTSD assistance dogs are service dogs trained to assist individuals living with PTSD. A growing body of research links the use of PTSD assistance dogs with substantial benefits for their human partners, including significant reductions in PTSD symptoms, and improvements in family functioning, social integration, and quality of life. However, research on the effect of assistance work on PTSD assistance dogs themselves is notably lacking. This paper aims to address this gap by mapping potential animal welfare and ethical concerns associated with PTSD assistance dog interventions. Moreover, a rights-oriented approach is employed, with the aim of suggesting guidelines that promote interspecies justice and contribute to the dogs’ well-being. The discussion highlights significant welfare concerns due to the lack of standardisation in the selection, rearing, training, and follow-up care of PTSD assistance dogs. Some of the symptoms and comorbidities common in people with PTSD (e.g., dysregulated anger, substance use disorder), along with some trained tasks of the dogs (e.g., diffusing the human’s episodes of distress/anger and interrupting flashbacks/nightmares), further exacerbate these concerns. PTSD assistance dogs also share a number of potential welfare issues with other assistance dogs, such as disruption of close relationships, lack of control over their physical and social environment, and insufficient down-time. To prevent animal exploitation and foster ethically desirable relationships with PTSD assistance dogs, their work conditions should not only minimise risks of harm but allow them to flourish and live a good life. Proposed guidelines include treating the dogs as agents, respecting their sustained dissent, providing sufficient rest, and allowing them to pursue their own interests. Furthermore, the work of PTSD assistance dogs should be enjoyable and beneficial for the involved canines, requiring force-free, non-aversive training and handling methods, and a strong bond with the human partner. Future research is needed to empirically investigate the welfare and ethical concerns highlighted in this paper, aiming to develop optimal practices that ensure PTSD assistance dog well-being.

## 1 Introduction

Posttraumatic stress disorder (PTSD) is a mental disorder manifesting as a lasting pathological stress response to an experienced/witnessed life-threatening or catastrophic event—such as combat exposure, physical/sexual assault, disaster (1). PTSD symptom clusters include intrusion (e.g., flashbacks, nightmares), avoidance of trauma reminders (e.g., places, people, thoughts), adverse changes in cognition and mood (e.g., emotional numbing, negative emotional state), and arousal/reactivity symptoms (e.g., sleep perturbations, hypervigilance, irritability, dysregulated anger, reckless/destructive behaviour) (1–4). In addition to physical health conditions (e.g., pain, musculoskeletal, gastrointestinal problems), various other mental health conditions (e.g., mood, anxiety, and substance use disorders) are common comorbidities (1, 3, 5–10). Thus, PTSD can be a debilitating disorder, dramatically hindering the person’s occupational, social, and family functioning and decreasing quality of life (1–4, 11–13).

Although the rates of PTSD vary across countries, it is often more prevalent among military populations—e.g., up to 23% of U.S. post-9/11 and 30% of Vietnam War Veterans (14, 15). In many societies, civilian rates also remain worrisome: e.g., lifetime prevalence among the general adult population is at 9.2% in Canada (16) and 7.8% in the U.S. (17). First-line treatments (pharmacotherapy and exposure-based psychotherapy)—although effective—show high drop-out and non−/under-response rates, and inconsistent outcomes (18–21). Consequently, non-exposure-based, less-stigmatising complementary and alternative treatments are emerging, including the increasingly popular assistance dogs.

PTSD assistance dogs are service dogs specifically screened and trained to assist an individual with PTSD (22). Since PTSD symptom profiles vary (1, 3), the trained tasks of assistance dogs differ according to their matched partner’s needs (9). Growing research links PTSD assistance dogs with substantial benefits for the human partner (23). These include clinically significant long-term symptom reductions (particularly intrusion/hyperarousal symptoms) (6, 24–27), and improved family functioning, social integration and quality of life (24–27). Demand for these dogs now exceeds supply—waiting periods for a fully trained dog can span years (25, 28, 29), with some providers even temporarily closing their application process (30).

Importantly, to date, virtually no published studies have examined how assisting a person with PTSD affects the canine partner of the dyad—a pattern consistent with the general state of human–animal interaction research (31). Ethical canine-assisted interventions should entail beneficial value, support individual interests, and reduce/avoid suffering for both partners (32–35). Considering the increasing number of assistance animals across human health conditions (34, 36), it is incumbent upon society to ensure the physical and mental well-being of these service animals and minimise their exploitation.

This paper maps the potential animal welfare and ethical concerns of PTSD assistance dog intervention to spur future empirical research. We adopt an ‘animal-rights’ rather than ‘welfarist’ ethical orientation (basically, a welfarist ethics amounts to an anti-cruelty ethic or one that emphasises being humane to other animals), holding that assistance dogs—like most humans^1^—possess rights. As with relevantly similar humans, these include fundamental negative rights not to be harmed, killed, tortured, confined, or, if appropriate, enslaved (37, 38), and the positive right to have a *good* life—to thrive. Based on this framework, we propose preliminary principles to guide efforts toward more just partnerships with PTSD assistance dogs, ensuring their work conditions allow them to flourish.

### 1.1 The variety of trained tasks of PTSD assistance dogs

Beyond the general benefits of dog-guardianship (e.g., social support, perceived unconditional love) (23, 27, 29), PTSD assistance dogs are particularly helpful due to their trained tasks. The top three most valued and used trained tasks (as reported by Veterans with PTSD assistance dogs) are calming during anxiety/distress, the positional cue “cover,” and interrupting or alerting to anxiety/distress, respectively (29). Episodes of anxiety/distress include intrusion and arousal symptoms, like panic attacks, nightmares, flashbacks, and uncontrolled anger (6, 9, 24–26, 29, 39). Assistance dogs interrupt with tactile distractions (nudging, pawing, licking) and calm the human through physical contact (leaning into, resting their head or laying on top of them) (6, 9, 29, 39, 40).

The “cover” task involves the dog positioning themselves behind the human on cue (29). This task is paired with another positional cue “block,” which sends the dog in front of the human (29). Both tasks instil a sense of security and ease hypervigilance in public as the human can rely on the dog to monitor approaching people and create space (9, 29, 41–43). When social interactions are desired, well-socialised and trained assistance dogs can facilitate the process by politely greeting people (e.g., sitting or offering a paw) (24, 29).

Further tasks include safety-checking rooms the person fears to enter (9, 24), and alerting to approaching people or strangers entering the home (25, 39). PTSD assistance dogs may also guide the person to a building exit during anxiety/distress episodes or to a designated location if disoriented on outings (26, 44). Additionally, dogs can be trained to refocus the person on their current task when distracted (26).

Depending on the individual’s needs, PTSD assistance dogs may be trained to retrieve or remind medication, wake the person when an alarm sounds, find someone nearby to request help, or contact emergency services/contacts via a K-9 speaker-phone or alert button (9, 40, 42, 44–47). For trauma survivors with mobility impairments, dogs can assist with bracing (supporting physical balance and stability), switching lights on/off, retrieving objects, opening/closing doors, and pulling wheelchairs (24, 42, 45, 46, 48, 49).

### 1.2 Lack of standardisation

A common challenge concerning assistance dogs, regardless of the human health condition they assist with, has been the lack of nationally recognized standardisation. Thus far, there are no Canadian or American federal standards for assistance dog selection, training, certification, or welfare (34–36, 50). However, both EU-wide and Australian national standards are under development, with the EU series at various stages of publication. In the absence of national standardisation, international bodies have played a central role: International Guide Dog Federation (IGDF) and Assistance Dogs International (ADI) are the two main organisations setting assistance dog training standards, certification criteria, and requirements for trainer qualification and animal welfare for their accredited non-profit members worldwide (34, 50, 51). While IGDF members provide assistance dogs exclusively for people with visual impairments, ADI members provide a wider range, including PTSD assistance dogs, which account for 18% of their dogs (52). However, high demand and low supply of assistance dogs have led to more dogs being acquired from non-accredited providers (34), fuelling a growing for-profit industry (53).

In an industry with minimal oversight, the price and quality of the services vary considerably. PTSD assistance dog training costs range from $15,000 to $35,000 USD in the U.S. (9, 24, 25, 47) and $3,000 to $50,000 CAD in Canada (35). As the certification process of both trainers and dogs has been unregulated, there is a considerable risk of receiving poorly trained dogs, or even becoming a victim of fraud, regardless of the hefty price tag (34, 53). For instance, a Canadian study by Vincent et al. (50) found significant inconsistencies in the selection, training, assignment, and follow-up of PTSD assistance dogs across seven schools (four non-profit, three for-profit), revealing that none fully met ADI criteria, certifications for dogs and trainers were absent, and overall adherence to ADI standards was low.

The absence of standardisation reflects the lack of agreement on optimal methods between the stakeholders (e.g., dog schools, Veterans’ organisations, policymakers, healthcare professionals) (50)^2^. This results in dog schools practising vastly different dog selection, training, and placement procedures that not only affect the efficacy and cost of this canine-assisted intervention, but also the welfare of the animals involved. Charities and other non-profit assistance dog providers are generally considered more reliable than for-profit organisations as they must comply with the mandates set by provincial, territorial, or state regulators (35) and are motivated to prioritise animal welfare as they depend on public donations (34).

## 2 Current PTSD assistance dog selection, training, and follow-up practices

### 2.1 Current practices of dog selection

The number of dogs who fail assistance dog training programs is notoriously high (54, 55). Studies report 40–50% drop-out rates to be common among guide dogs (54, 56). Similar attrition rates apply to PTSD assistance dogs (57), contributing to their low supply. Although the dogs’ physical health problems can lead to drop-outs, behavioural issues—particularly, aggressiveness, shyness, and fearfulness—are the predominant reasons (36, 54, 56, 58). Considering the financial and time costs of training assistance dogs (36, 55), it is paramount for agencies to carefully select the dogs who enter their program. However, in the context of a scarcity of studies on the topic, the selection processes vary widely depending on the agency.

Agencies training PTSD assistance dogs differ in terms of whether they source their dogs from animal shelters or selective breeding programs (6, 39, 41, 45, 46, 49). For instance, K9s For Warriors (the foremost U.S. provider of PTSD assistance dogs) advocates for sourcing dogs from animal shelters, identifying as “the world’s largest rescue to service program” (59). It is only since the COVID-19 pandemic (in 2022) that they began additionally procuring animals through breeding programs, donations and guardian surrenders to reduce their then four-year waitlist. Still, 60% of their dogs are rescues (59). When it comes to donated or surrendered dogs, K9s For Warriors accepts dogs of various ages: 8 weeks–3 months and 11 months–4 years (59).

Another major U.S. PTSD assistance dog provider, Warrior Canine Connection (WCC), has a different approach, as they rely on their own breeding program, selectively breeding dogs for temperament, physical health, and longevity (49, 60). They evaluate dogs’ pedigree across 12 generations to screen for individuals with genetic potential for PTSD assistance dog work. In terms of temperament, they select for dogs who have minimal prey drive, do not exhibit aggressive behaviours, are not easily aroused, are affectionate and eager to please, and possess high sensitivity to human emotions (49, 60). The agency puts great care into puppy socialisation to ensure their assistance dogs will facilitate social interactions for their human partner. Similarly, great care is put into safely and gradually preparing the young puppies for experiences and environments involved in their future occupation (49, 60).

The fact that both K9s For Warriors and WCC are ADI-accredited yet have vastly different selection processes underscores, again, the lack of agreement on best practices for maximising the number of graduating dogs and successful recipient-dog teams. These two sourcing approaches are both common among PTSD assistance dog providers. In the abovementioned Canadian study by Vincent et al. (50), four schools received dogs from shelters and through donations, while three had their own breeding programs. A third, less common option is for people with PTSD to have their own companion dogs trained as assistance dogs (25, 34, 35, 53). While some praise the use of shelter dogs or companion dogs as financially more affordable options (25, 35), others warn against the higher risk of these dogs not passing the training program (36, 43).

Likewise, no consensus has been reached regarding breed preference. WCC breeds exclusively Golden and Labrador Retrievers (60). A study recruiting dogs from K9s For Warriors also reported the majority of the rescued dogs being Golden and Labrador Retrievers or mixed breeds (46). Furthermore, Vincent et al. (50) reported Labrador Retrievers as the most common breed among the investigated Canadian PTSD assistance dog schools. This is consistent with the rest of the assistance dog industry, where Labrador and Golden Retrievers, or cross-bred retrievers have been the breeds of choice for most assistance dog jobs (apart from hearing dogs) (51, 56, 61–63). Nevertheless, neither ADI nor the European standards restrict breed choices (61, 64) and the preferences among PTSD assistance dog schools can vary from “all breeds except large dogs and those with a bad reputation” to only “large working breeds” and from “Labrador, Labernese, Golden Retriever, Labernese mixed with Golden Retriever” to “all breeds except Labradoodle and bully breeds” (50, p. 34).

It is reasonable to assume that the main motivation behind excluding large breeds or breeds with poor reputation is at least partly because these dogs can be perceived as intimidating. After all, PTSD assistance dogs accompany their human partners to public places and are supposed to facilitate social interactions. Indeed, participants in an American study investigating people’s perception of service dogs of different breeds reported feeling uncomfortable around Pit Bull-type dogs (due to the mostly unfounded belief that these dogs are more aggressive) (65). There are also convenience- and practicality-related factors: it is easier to bring smaller dogs to certain public spaces (e.g., cinema, restaurant, plane) and to house them in small apartments. However, in case of recipients who also need mobility support from the dog (e.g., wheelchair-pulling, physical bracing, opening doors or retrieving objects), opting for a large breed is necessary. In fact, it has been argued that Golden and Labrador Retrievers are the preferred breeds for many assistive tasks predominantly due to their optimal size, not behaviour (54, 63). However, the Golden and Labrador Retrievers’ non-intimidating image that makes people feel comfortable around them (65) and—for those requiring retrieving assistance—their gentle grip when carrying objects (66) are further assets. All things considered, matching assistance dogs with recipients requires careful consideration, and the breed size and type largely depends on the specific needs, preferences, and lifestyle of the assisted individual (46, 50, 54).

The same situation of varying practices applies to temperament screening (46, 50). Whether PTSD assistance dogs are procured from shelters, through donations, or specifically bred, they are all screened for temperament (25, 46, 50). Again, the selection criteria vary between organisations (50). The most commonly desired traits in PTSD assistance dogs overlap with, but also extend beyond, those bred for by WCC: sensitivity toward human emotional states, lack of aggressive behaviours, low prey drive, low distractibility, high stress tolerance, and not being anxious, fearful, or easily startled (36, 50, 56). Proactiveness is a further desired trait (50) as some PTSD assistance dogs’ trained tasks require taking initiative (e.g., removing the human from the situation when they get disoriented).

While these traits are not always formally evaluated (50), available assessment methods include standardised and non-standardised behavioural tests, evaluations of external factors (e.g., maternal care), behavioural lateralization, physiological/neurohormonal profiling (e.g., cortisol and immunoglobulin A), and questionnaires for trainers/puppy-raisers (36, 56, 67). In the absence of uniformly recognized selection procedures, the choice and interpretation of the results of such assessments differs (50, 63, 67). Moreover, there is a considerable gap of knowledge on the suitability of these assessment methods as tools predictive of assistance dog success (36).

Using external breeders and/or Golden and Labrador Retrievers, or their crosses, has sometimes (but not always) been linked to training/working success (36, 68–70). Nevertheless, the selection of breeds tested is narrow and evidence for breed-based discrimination in assistance dog temperament screening is mixed and inconclusive. A Swedish study by Svartberg (71) of over 13,000 dogs from 31 breeds found Labradors and Flat-coated Retrievers highly social, curious, and fearless, while Golden Retrievers, though social, were among the least curious and fearless. Surprisingly, Labradors also ranked among the more aggressive breeds (71). Turcsán et al. (72) analysed 5,700 guardian surveys on 98 breeds, categorising Golden and Labrador Retrievers as sociable, bold, calm, and moderately trainable, with Flat-coated Retrievers and German Shorthaired Pointers ranking even higher. Since sociability, calmness, boldness, trainability, and low aggression are desired traits in PTSD assistance dogs, some studies thus endorse favouring retrievers. However, it must be considered that a dog’s personality develops through a combination of genes, early life experiences, hormones, and environment, and within-breed variability is similar or even higher than between breeds (71–73).

Historically, dogs were bred for functional purposes (e.g., herding dogs, guard dogs, hunting dogs) (73), creating breed groups with genetic breed-group-typical behaviours: spitzes/“primitive-type” dogs, sled dogs, sheep/cattle dogs, terriers, scent hounds, sight hounds, retrievers, and pointing dogs (74). Hence, there is evidence that ancestry has an influence on behaviour as breed groups vary in behavioural phenotypes and certain propensities, including temperamental motivation to perform specific challenging tasks, proneness to being fearful/anxious, and willingness to take direction from humans as opposed to being more self-directed learners (73, 74).

Nevertheless, it is unclear how much these historically bred functional traits persist in contemporary breeds that, having started radiating only during the Victorian era, are younger than 160 years, mostly defined by aesthetic features, and often disconnected from their original work-related selection (73). An extensive study by Morrill et al. (73) analysed the behaviour and ancestry of over 18,000 dogs and sequenced more than 2,100 dogs’ DNA. The authors reported that breed explained only about 9% of the variations in dog behaviour, whereas the association between breed and the likelihood to exhibit aggressive behaviours was particularly weak (73). In summary, although behaviour patterns are breed-specific (especially in working-line breeds), breed is a relatively poor predictor in terms of behavioural traits (such as aggressiveness).

Another topic of concern is selective breeding. There is substantial ambiguity of trait heritability—i.e., how much the variability of a certain trait within a breed, population, or a group depends on genes (58). Measuring trait heritability is challenging, requiring well-defined traits, large sample pools with sufficient variability of that investigated trait, and reliable methods. This area of research is notoriously complex and yields mixed results. For instance, aggressiveness heritability ranges widely across literature (h^2^ = 0.06–0.77) (75, 76) and varies by breed, with Golden Retrievers showing higher heritability for stranger-directed aggression (h^2^ = 0.77) than Labradors (h^2^ = 0.29) (75).

Reviews of the existing literature on traits relevant to assistance dogs have reported general fearfulness as the main heritable temperamental trait—and the most common reason for failing training programs (36, 58). Other traits investigated among assistance dogs, albeit not always predictive of training success, have ranged from non-heritable to low (e.g., noise sensitivity, restraint tolerance) or moderate heritability (e.g., following when called, retrieving response) in breeding stocks of Golden/Labrador Retrievers and their crosses (36, 70). While highly heritable traits like fearfulness vary a lot between individuals and can be effectively adjusted through direct selection, traits with low variability are less responsive to breeding unless progeny selection or crossbreeding is implemented (58). More research is required to identify proclivities predictive of success in PTSD assistance dogs and their heritability in different breeds/populations. Until then, a case-by-case selection remains the best strategy.

### 2.2 Current dog training and follow-up practices

The next step after a dog has been selected for a PTSD assistance dog program is training. Examples of common training phases include basic skills/obedience training, advanced/customised skills training, and public access certification training, while pair-training^3^ with the matched person is usually (but not always) the last phase (50). Although ADI sets some standards, they leave room for interpretation, reflecting the lack of scientific knowledge on the best training practices in assistance dogs (36). According to the summary of ADI standards (77), assistance dog training programs are required to follow consistent high-quality dog selection and training procedures that are comprehensive and individualised to meet the needs of both the recipient and the dog. ADI has developed their own Public Access Test to ensure that all ADI-accredited agencies train their dogs to be well-mannered, composed, inconspicuous, and never unruly in public settings. In addition, the dogs need to be house-trained, not exhibit aggression, and possess a minimum of three trained assistive skills specific to the recipient’s disability (77). Similar requirements are articulated in the newly published European standards (64). The means to meet these criteria can vary considerably from school to school. For instance, Vincent et al. (50) noticed that, while most of the seven studied schools did not conflict with ADI criteria, the schools practised different training methods, trained different tasks to PTSD assistance dogs, and dedicated varying amounts of time to their various phases of dog training and pair-training.

The training duration also depends on how the dogs are sourced. For example, K9s For Warriors provides their rescued dogs with 6–8 months (minimum of 60 h) of preparatory training, followed by 2–3 weeks of pair-training (6, 41, 45, 46, 59). In contrast, dogs born in the WCC’s breeding program undergo about 2 years of training (60), culminating in 7–8 days of pair-training (WCC, personal communication, April 26, 2024). ADI-accredited organisations accepting dogs trained by guardians or private trainers must work with those guardian/trainer-dog teams for at least 6 months before certification (77).

Vincent et al. (50) found training periods ranging from 8 months to 2 years, including pair-training which lasted from 1–13 months and commenced anywhere between the 2nd and 22nd month. Yarborough et al. (39, 47) also reported considerable variation among five U.S. non-profit providers (four ADI-accredited), with pair-training formats ranging from two-week intensive camps to multi-session seminars or trainer-determined case-by-case timelines (39, 47). In contrast, Audeamus, Inc., a Canadian non-profit (not ADI-accredited), employs a “hands-on” model where the entire training process (i.e., from basic skills to public access training) involves pair-training—Veterans train assistance dog candidates throughout a year-long program under professional guidance (35). While this approach aims to strengthen the Veteran-dog bond, dogs typically still require 2 years of training before being granted public access (35).

Likewise, there is no consensus across organisations regarding the skills—whether basic obedience or assistive—taught to PTSD assistance dogs. Assistance dogs in a study by Saunders et al. (42) were trained to perform five PTSD-specific tasks. Vincent et al. (50) reported five basic trained tasks and six PTSD-specific trained tasks, although 2/7 schools involved no basic training and one taught no PTSD-specific tasks to their dogs. Lessard et al. (26), who recruited participants from four Canadian PTSD assistance dog schools, also noted that some schools do not teach PTSD-specific tasks but focus solely on nurturing the recipient-animal bond, considered fundamental for the dog’s natural (i.e., untrained) assistance during intrusion/arousal symptoms. This prompts the question of how assistance dogs without trained disease-specific skills differ from companion dogs (including emotional support dogs). On the other side of the spectrum, are schools training dogs to perform a minimum of 70 (47) or even over 80 tasks (60), although it is unclear how many are PTSD-specific. Contributing to the diversity of approaches, some agencies teach the same task repertoire to every PTSD assistance dog, while others follow a tailored approach and train the dogs according to their matched person’s needs (50).

Absence of consistent practices is also evident in training principles. The summary of ADI standards states that dogs should be trained by humane and evidence-based methods, and the least aversive and intrusive techniques should be preferred (77). The newly published European standards provide even clearer guidance, mandating positive reinforcement-training and prohibiting aversive equipment and techniques (64, 78). However, again, organisations have demonstrated varying interpretations of ADI guidelines. Some agencies, such as WCC and Audeamus, exclusively utilise training methods consistent with positive reinforcement-training (35, 49, 60), while others include aversive techniques. In Vincent et al. (50), 2/7 PTSD assistance dog schools used choke/tone collars, and one considered pain application as a less common dog training method.

LaFollette et al. (79) investigated training methods that K9s For Warriors taught to Veterans with PTSD and that the Veterans were instructed to use after bringing their assistance dog home. They identified five main training styles: positive reinforcement (physical/verbal praise, play/food reward, clicker-training), negative punishment (ignoring the dog’s unwanted behaviours, time-out), positive punishment (flat/prong collar correction, physical/verbal correction), dominance-based interaction (eating before dog, alpha-roll, stare-down), and bond-based interaction (co-sleeping, sharing food, “do as I do”) (79). The 111 Veterans in the study reported using methods of all these categories once a month or more. The most common were positive reinforcement techniques, which almost all participants used daily. Positive punishment was the second most common method with verbal and leash corrections used daily by most participants. These were followed by bond-based interaction, with 50% of the Veterans co-sleeping with their dog (likely to tackle nightmares), and then dominance-based methods, of which eating before the dog was the most common (used by 38% of Veterans). Ten percent of Veterans reported daily use of negative punishment (79).

Regarding post-training routines—e.g., follow-ups to ensure the human-dog dyad functions in a manner conducive to both partners’ welfare—ADI requires programs to provide support for the dog’s lifetime through in-person visits or remote contact (50, 77). These include follow-up contacts within a month after placement, an at-home visit 3 months after placement, minimally at every 3 months during year one of placement, and minimally once a year after that (50). In addition, ADI-accredited organisations must collect annual veterinary reports and allow for emergency or unplanned follow-up care upon recipient’s request (ADI, personal communication, April 16, 2024). Similarly, the newly published European standards require annual veterinary examinations and regular instructor visits to assess the dog’s ongoing fitness for work (78).

Needless to say, the follow-up routines vary, particularly among non-accredited organisations. Many for-profit agencies discontinue their involvement entirely once the dog is sold (53). Vincent et al. (50) reported some schools providing no follow-up care, while others offered it only upon request. Although there were also rigorously structured follow-up schedules, none met ADI’s optimal criteria (50).

Follow-up care should also include maintenance training to ensure dogs continue to perform their trained skills over time. ADI regulates accredited organisations to provide monthly maintenance training during the first year and as needed thereafter, with a minimum of once a year during recertification (WCC, personal communication, June 12, 2024). ADI-accredited organisations must recertify PTSD assistance dog teams annually via the ADI Public Access Test (WCC, personal communication, April 15, 2024). Maintenance training and recertification are unregulated among non-accredited providers. For instance, Audeamus recertifies their teams once every 3 years (35).

Currently, there exist no standardised evidence-based protocols for the retirement of assistance animals (51). The decision to retire an animal is multifactorial and highly individual, based on the needs of each dog and team (51) (WCC, personal communication, April 15, 2024). Therefore, neither ADI nor the European standards dictate a strict age by which dogs must retire or enforce mandatory retirement standards (besides annual follow-ups and veterinary reports that, among other considerations, address this issue; ADI, personal communication, April 16, 2024) (78). The retirement process looks different even among ADI-accredited organisations (51). WCC has developed guidelines to help recipients look out for their assistance dog’s age-related changes that warrant discussions around retirement (WCC, personal communication, April 15, 2024). When agencies transfer dog-ownership entirely to handlers, or private trainers train companion dogs as assistance dogs, there is no oversight regarding the dog’s retirement (51).

## 3 Fostering PTSD assistance dog well-being: welfare concerns and ethical guidelines

The lack of standardisation in the selection, training, certification, and follow-up care of PTSD assistance dogs, some PTSD symptoms/comorbidities, and some PTSD-specific tasks dogs are expected to assist with, raise reasonable concerns about animal welfare. Reputable agencies—particularly ADI-accredited ones—promote humane care of assistance dogs (34, 77, 80), e.g., by mandating regular veterinary inspections, follow-up care, and recertifications. Agencies that retain legal ownership of the animal after placement can reclaim dogs in poor condition (80). However, few go beyond merely preventing suffering to actively ensuring that the dogs live *good* lives—that they thrive (34).

The assistance animal field is notably anthropocentric. Laws and policies safeguard the rights and quality of life of the human partners but overlook those of the animals (34). Research on the welfare of assistance dogs—particularly psychiatric, mobility, and hearing dogs—is sparse (34, 36, 63). Although the need to investigate PTSD assistance dog welfare has been voiced (25, 35, 40), thus far, this area of research remains undeveloped (34).

Public perception often assumes assistance dogs enjoy better welfare than companion dogs (34, 54, 81). For instance, research conducted among 258 Australians reported most people considering assistance dogs happier due to their constant companionship, strong human-animal bond, high-quality care, and presumed meaningful work (81). Nevertheless, concern is growing around ethical issues including stressful/restrictive work conditions, harsh training, limited rest, and the ability of some disabled people to meet their dog’s needs (81). Indeed, according to canine behaviour and welfare researchers, Ray Coppinger and James Serpell, assistance dogs often endure stressful occupations with little self-agency or benefits from their good performance (54, 63).

As the renowned Five Freedoms^4^ advance into richer models, scholarly views of animal welfare are also evolving. While historically, animal welfare focused on physical health concerns/stressors (34, 82–84), increasingly favoured contemporary approaches consider animals’ physiological, psychological, and emotional states (82–85). In this contemporary sense, animal welfare refers to the quality of an individual sentient animal’s life as determined by their subjective experiences—i.e., states the animal experiences as positive or negative (83, 84).

A criticism of traditional animal welfare addresses its preoccupation with reducing negative states, with insufficient attention to encouraging positive states (31, 34, 35, 82–84). Historically understood, welfare thus has a negative connotation that something is missing or needs fixing (82, 83). To bring attention to a more positive connotation, the term ‘well-being’ can be favoured in the discourse (although many speak in terms of negative and positive welfare). Here, well-being refers to an animal’s state of health, comfort, and *happiness*—emphasising positive experiences and the capacity to thrive (31, 35, 82, 83, 85). These more positive orientations inform the Five Domains Model^5^, which builds on the Five Freedoms by promoting positive experiences (in addition to minimising negative experiences) and addressing animals’ emotional/psychological needs (82, 84, 86). Notably, the recently published European standards have also adopted the Five Domains Model and place explicit emphasis on promoting assistance dog well-being (64, 78).

In what follows, we adopt a particular ethics-oriented approach that goes beyond merely preventing the animals’ “unnecessary” suffering and meeting their most important needs. We will first identify key welfare concerns for PTSD assistance dogs (what appears lacking and needs fixing) and then explore further moral considerations for supporting their well-being—ensuring they live *good* lives, and their work is enjoyable and creates beneficial value to them. Core principles of our approach include respecting animal agency, opposing forced labor, and supporting the dogs’ own interests.

### 3.1 Potential welfare concerns of PTSD assistance dogs

Numerous factors may compromise PTSD assistance dog welfare—some common to all assistance dogs, others PTSD assistance dog-specific. The first category we canvass comprises aspects of a typical assistance dog’s lifecycle, rearing, selection and training methods, which, as already described, vary across organisations. The second involves the nature of PTSD assistance dog work, including work conditions, trained tasks, and exposure to certain PTSD symptoms and comorbidities that might create a hazardous environment for the dog.

#### 3.1.1 Typical lifecycle of PTSD assistance dogs

**Figure 1:**
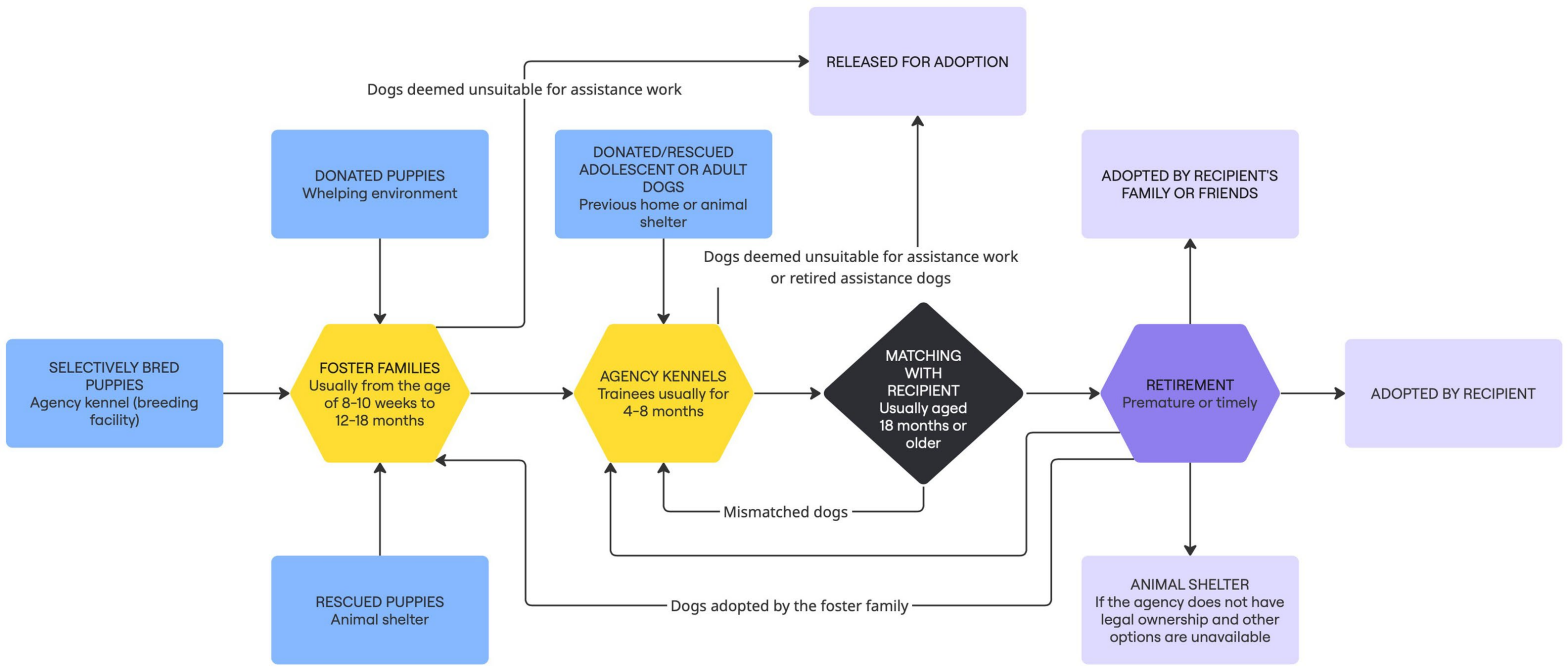
Typical lifecycle pathways of PTSD assistance dogs. Companion dogs privately trained for assistance work are not included.

Assistance dogs (including those for PTSD) undergo several major life transitions that disrupt their physical and social environments (54, 55, 62, 63) (Figure 1). Selectively bred assistance dogs or those sourced as puppies are typically placed with foster families at 8–10 weeks for 12–18 months (51, 54–56, 59, 62, 63, 87). With different degrees of agency oversight, the foster family is responsible for socialising the puppy, exposing them to a wide range of public places/situations, and teaching them basic obedience (55, 62, 78, 87). Upon reaching adolescence, dogs return to agency kennels, where they are housed in stalls (often individually) over the period of advanced training, culminating in pair-training (36, 54–56, 59, 62, 63). The length of this kennel-life period varies from 4–8 months (54, 56, 62). Dogs sourced outside the breeding programs as adolescents/adults (e.g., rescued/donated dogs) likely spend their full training time at the kennel.

Some evidence suggests 17 months is the optimal age for transitioning back to kennels (55), allowing time for certain genetic or behavioural issues to manifest (54) and agencies to decide on further training investment. Although using puppy-raisers and the subsequent advanced training at kennels is common among ADI-accredited PTSD assistance dog schools, specific practices vary. For instance, WCC’s dogs live with puppy-raisers until 21 months, followed by 3–4 months of kennel-based advanced training and a week of personalised training with the recipient (WCC, personal communication, April 25–26, 2024).

Dogs are usually placed with recipients at 18–24 months of age (54, 55, 62, 78), unless enrolled in the program later. Being matched does not necessarily mean the dog has found a long-term home. Assistance dogs have been relinquished due to temperament mismatch, behavioural issues, or handler-related circumstances (34, 35, 62). Unfortunately, there is also evidence for an instrumental view of assistance dogs—among recipients who treat them merely as tools for health-related goals. Williamson et al. (35) reported a case where a Veteran, despite bonding with their PTSD assistance dog, relinquished the dog after a year, feeling the dog’s support was no longer needed. If legal ownership remains with the agency, such dogs may be retrained and/or reassigned (35, 62). Dogs deemed unsuitable for assistance work are retired prematurely (62).

Retired assistance dogs may be adopted by their handler or handler’s family/friends, return to the puppy-raiser, or go back to the agency to be released for adoption (34, 51, 62, 63). Dogs who drop out of training follow similar paths (88, 89). However, when a dog was privately trained, or agencies transfer legal ownership completely to handlers, and no family/friends can adopt the dog, the retiring assistance dog might be relinquished to an animal shelter (34). While their training and temperament typically make assistance dogs desirable for adoption (51), euthanasia is possible for those not placed in a no-kill shelter.

Such a succession of families and uprooting from familiar environments can negatively affect a dog’s welfare (34, 54) and potentially foster behaviour problems that hinder future performance (63). These could include separation anxiety, excessive barking/vocalising, hypervigilance, destructive behaviour, inappropriate elimination, and difficulty focusing during training/working (90). Such disruptions can be especially stressful for assistance dogs, who are typically selected for their tendency to form strong attachment bonds with humans—and may be particularly vulnerable to separation anxiety (91). Although the exact impact of repeatedly breaking these bonds is unclear, many experts argue it can be deeply distressing (32, 54, 63).

Mengoli et al. (56) found that assistance dogs had significantly lower free and total oxytocin levels than companion dogs, potentially due to repeated separations from foster families during kennel-training period (characteristic to their setting). It was suggested that such social disruptions alter dogs’ bonding-related neuromodulatory system, negatively affecting future attachment processes (56). Conversely, Hall et al. (55) encouraged foster families and trainers to bond with dogs, suggesting that secure and high-quality attachment bonds in earlier life facilitate developing bonds later and thus positively impact the dogs’ well-being and performance after placement. Attachment to foster families is also encouraged in the European standards (78).

Varying personalities, expectations, and training/interacting styles among foster families and handlers further challenge the dogs, especially when placed with inexperienced people (34, 35, 54, 63, 92). Poor training and handling skills can confuse dogs and reduce their performance, affecting the handler’s health benefits and satisfaction (63), and potentially lowering their commitment to the dog’s well-being (80). Although pair-training includes developing recipients’ dog-training/handling skills, the varying lengths of pair-training across agencies (from a week to 13 months as reviewed above) raises serious welfare concerns for PTSD assistance dogs.

Major life changes, like moving to a new home, can be stressful for a dog even when remaining in the same family (93). Drastic changes to the dog’s physical and social environment—like moving from family home to kennel—can be even more challenging, particularly for dogs who did not experience kennel life during early puppyhood (54, 94). Life in kennels varies across organisations. While some agencies incorporate training sessions, outings, playgroup-time, and enrichment activities into the dogs’ schedule (WCC, personal communication, April 25, 2024), others may take the dog out of the stall for only 20–60 min each day (54) and provide little to no enrichment (63).

Kennel stress is not an uncommon reason for assistance dog candidates to drop out of programs, just like it is not uncommon to develop behavioural issues during kennel stays (54). Although ADI standards emphasise the importance of enrichment during kennel housing (77), kennels remain impoverished environments—even at top-tier agencies (54, 94, 95). Loud, crowded, unfamiliar, and understimulating kennel environments can elicit stress, (separation) anxiety, stereotypic behaviours (e.g., pacing, excessive barking), as well as hyperactivity, fearfulness, and dog-directed aggression (63, 94–97). It goes without saying that similar welfare concerns apply to relinquished/retired assistance dogs in animal shelters.

#### 3.1.2 The role of early life experiences in PTSD assistance dogs’ welfare

Rescuing a dog from a shelter offers obvious welfare benefits (admittedly, depending on the contrast between the shelter and the new home). However, such dogs have been subjected to kennel conditions and may have developed above-described issues that could compromise their future performance and welfare as assistance dogs. Shelter-sourced dogs are carefully screened, but the lack of validated screening procedures and the latent nature of many behaviour problems (63) remain major challenges. Their unknown background—particularly during early puppyhood—is another concern. Hence, critics argue that organisations using shelter dogs risk providing animals unsuitable for assistance work (35, 36, 43, 63).

Early experiences during the sensitive period of socialisation—typically between 4 and 16 weeks in dogs—are critical for behavioural development (54, 55, 63, 90, 98–101). During this time, puppies are prone to bond easily and accept novel social and environmental stimuli (98), building lasting associations that are difficult to unlearn later in life (63, 98, 101). Prospective assistance dog puppies must be introduced to a wide range of potentially fear-provoking stimuli related to their future occupation (including various people, animals, locations, sounds) in a safe and positive way to shape their perception of what is “normal” in their environment and prevent future undesirable responses (54, 55, 63, 64, 78, 98, 101).

Shelter dogs’ unknown background entails the possibility of negative or insufficient exposure during the socialisation period or growing up in an environment not conducive to learning to cope with novelty. This may lead to fearful or aggressive responses to novel or negatively associated situations/stimuli (63, 98, 101)—common reasons for dogs failing assistance dog training. In contrast, breeding programs can provide controlled environments and structured socialisation to ensure the dogs learn to handle relevant stimuli and novelty with calm and confidence (55, 60, 63, 77). Moreover, some suggest that positive associations formed during proper socialisation may boost the dog’s motivation to work (55) and make the work more enjoyable, as the dog could develop cognitive abilities to better understand the tasks’ purpose and rely less on operant conditioning (63).

WCC’s Puppy Enrichment Center supports early socialisation (87). Additionally, they involve Veterans with PTSD in socialising and training future assistance dogs for fellow Veterans—both to prepare the puppies and provide occupational therapy for the Veterans (25, 45, 49). Nevertheless, not all assistance dog breeding kennels offer adequate socialisation opportunities (54, 63), setting dogs up to struggle with future work-related demands. After all, the “flunk-out” rate is high among agency-bred dogs alike and impoverished early environments are a contributing factor (54, 63). In such cases, transferring puppies to foster homes earlier can help. However, if agencies provide sufficient enrichment and outside exposure, keeping puppies with their mother until 12 weeks supports social learning (by observing her performance), with a to-be-determined benefit for the pups’ training outcomes (55).

Further factors that contribute to the high drop-out among agency-bred dogs and lifelong welfare concerns are genetic health issues (e.g., progressive retinal atrophy, osteoarthritis), inbreeding, susceptibility to infectious diseases, and other problems associated with a narrow gene pool (36, 54, 63). Along with uncertainties around trait heritability and breed preferences, this raises doubts about whether breeding programmes of solely pure-bred lineages are actually desirable for this type of work (36, 63).

Thus, while some evidence links selectively bred assistance dogs to better training/working success (36, 68, 70), these benefits can be undermined by organisational practices and conditions. More research is needed to lower failure rates and improve dog welfare—such as comparing welfare and return rates of shelter-sourced vs. purpose-bred PTSD assistance dogs and examining recipient health outcomes. Recommendations have included exploring out-crossing and crossbreeding to boost the health of selectively bred stocks, while also highlighting the need for better behavioural screening methods, particularly for identifying suitable shelter dogs (63, 102). For instance, Mengoli et al. (56) suggest that neurochemical profiling (e.g., prolactin, oxytocin, serotonin) could enhance assistance dog selection, breeding, and welfare monitoring.

#### 3.1.3 The use of aversive techniques in PTSD assistance dog training and handling

Despite ADI’s recommendation to use humane, evidence-based, minimally aversive training methods (77), as discussed earlier, aversive and dominance-based methods are relatively widespread among PTSD assistance dog schools—even some ADI-accredited ones. The use of dominance-based methods contradicts ADI standards, as dominance theory within the context of human-dog relationships is outdated and lacks scientific support [see (103–106) for explanations for dismissing the dominance-based approach]. Insurmountable evidence shows that dominance-based human-dog interactions (e.g., alpha-rolls, stare-downs) induce fear in dogs, increase handler-directed aggression, and damage dog-handler relationships by undermining trust, emotional bonds, communication, and cooperation (90, 99, 100, 104, 107).

The use of aversive stimuli within positive punishment and negative reinforcement can be considered evidence-based when understood as integral principles of operant conditioning. However, it is important to consider *all* the evidence, including the growing research highlighting their harm to dogs’ physical and mental health (36, 54, 55, 63, 90, 100, 107, 108). Aversive methods can also elicit unwanted behavioural side-effects, hinder performance and motivation, and compromise dog-handler relationships (36, 55, 90, 99, 100, 107–109).

Ziv’s (107) review of scientific studies on dog training methods shows positive punishment and negative reinforcement often increase dogs’ fear (including fear of novelty and punishment-related stimuli), aggression (toward people/dogs), stress (e.g., stress-indicative body language, restlessness, cortisol levels), distractibility, escape/avoidance behaviours, hesitance to explore, apathy/passiveness, and hypervigilance. These outcomes—especially heightened fearfulness, aggression, stress, and anxiety—are consistently documented by scholars and practitioners (55, 63, 90, 98–100, 104, 110).

Aversive techniques linked to negative behavioural and psychological effects include verbal (e.g., yelling “no,” scolding), physical (e.g., kicking, hitting, shaking, scruff-grabbing, alpha-roll), and collar corrections (e.g., choke, tone, shock, prong, spray collars, or yanking by a flat collar) (107). Many also pose physical health risks. These involve injuries from kicking or forced restraint (e.g., alpha-roll), and damage from punitive collars—including increased intraocular pressure, spinal cervical injury, nerve damage, esophageal/tracheal injuries, and impaired respiration (100, 107). Stress-induced concerns, like gastrointestinal problems and weakened immunity add further risk (107).

Supporters of aversive training argue that, when used professionally, such tools do not harm dogs. However, Ziv (107) found negative welfare impacts even when applied by experienced trainers. Risks increase—potentially reaching abuse—when used by unskilled handlers lacking timing and consistency (107). With no trainer qualification standards, recipients’ general inexperience, and brief pair-training, aversive techniques pose significant welfare concerns for assistance dogs. In contrast, positive reinforcement-based training carries far lower risks of behaviour problems, aggression, and fear—even when applied inexpertly (100, 107).

Considering that fearfulness and aggression are highly undesirable in PTSD assistance dogs and top reasons for training failure, it is regrettable that some agencies’ methods contribute to these problems. Even when dogs complete such programs, the harm and suffering caused remain. Moreover, research shows punitive techniques often reduce dogs’ willingness to approach strangers and interact/play with handlers (107). The latter reflects damage to the dog-handler relationship due to dogs associating handlers with aversive stimuli (99, 100, 104, 107).

There is a major research gap on how different training methods affect assistance dogs specifically (36). However, LaFollette et al. (79) found similar outcomes: PTSD assistance dogs of Veterans prone to use positive punishment were more fearful, less active, and less playful than dogs of Veterans using positive reinforcement-based techniques. Moreover, frequent positive punishment use was linked to weaker perceived Veteran-dog bonds and less eye contact, while positive reinforcement and bond-based interactions were associated with stronger bonds (79).

As discussed, sociability with strangers is highly desirable in PTSD assistance dogs and the recipient-dog bond is crucial for recipient health benefits (29). Thus, aversive methods should be impermissible when training/handling PTSD assistance dogs—both because they harm dog welfare and undermine training goals. Moreover, positive reinforcement-based methods promote handler patience, attention, emotional regulation, and expression of positive emotions (e.g., when praising the dog), mitigating PTSD symptoms like emotional numbing, avoidance, and hypervigilance (49). Hence, they benefit both the dog and the person with PTSD.

Certain situations may require assistance dogs to take initiative or disobey commands (“intelligent disobedience”) (54, 55, 64). Encouraging this is incompatible with associating disobedience with aversive stimuli (55). Exposure to aversive stimuli perceived by the animal as inescapable and/or unpredictable (e.g., due to inconsistent or poorly timed punishment) can cause the animal’s learned helplessness (100, 104, 110), manifesting as passivity, and impaired decision-making and initiative-taking (7, 111, 112).

Some tasks expected of assistance dogs (including some PTSD assistance dogs)—e.g., wheelchair-pulling—lack discrete beginning/ending, might be physically distressing, are not intrinsically motivating, and the dog may not fully understand their purpose (54, 63). In such cases, it is sometimes argued that positive reinforcement-based techniques are impractical for meeting training goals and maintaining performance levels after placement (63, 92). Although none of the studies reviewed by Ziv (107) analysed wheelchair-pulling *per se*, no evidence indicated that aversive methods are more effective. In fact, reward-based training is linked to increased motivation, improved performance (e.g., in obedience, protection, scent-detection) (107, 108), and higher trainability (79). Incorporating punishment in training complex tasks can make them even more aversive, further reducing the dog’s willingness to work, and triggering avoidance and other unwanted side-effects. Tasks requiring the most stamina and motivation are precisely those expert trainers avoid associating with punishment (63).

Hopefully, the prevalence of aversive methods will decline substantially, at least in Europe, now that the new EU-wide standards explicitly prohibit their use (64, 78). Non-aversive alternatives should be explored where positive reinforcement falls short. Hall et al. (55) suggest investigating Pavlovian-conditioned reinforcers (e.g., clickers) for sustaining performance when primary reinforcers (e.g., treats) are impractical. If applied appropriately, gradually introducing intermittent reinforcement schedules can also increase resistance to extinction and reduce frustration from inconsistent reinforcement in real-world conditions (55, 113–115). However, research focused on such non-aversive solutions is still lacking.

#### 3.1.4 Welfare concerns related to the nature of PTSD assistance dog work

Bremhorst and colleagues (36) noted that agencies still prioritise assistance dogs’ physical health, while psychological/emotional welfare often receives scant attention. Several researchers as well as the European standards urge a broader assistance dog welfare approach aligned with the Five Domains model. One general concern is that—due to limited knowledge or financial restraints (34, 116)—recipients may not always provide adequate canine care despite education during pair-training (35, 42, 47). Further concerns stem from the nature of assistance dog work: insufficient rest, constant work, unpredictable routines, limited play/recreation opportunities, unintentional maltreatment, zoonotic disease risk, and even obesity (34, 36, 51, 62, 63, 117). Another issue is assistance dogs’ inability to control their physical and social environment or escape stressful, potentially dangerous situations (63, 117). Examples include busy streets and crowded public spaces where avoiding unwelcome interactions is difficult, and home settings where contact with non-recipient family members may be restricted (62).

While PTSD assistance dog recipients have reported frustration from the dog’s around-the-clock company (35), it is unclear whether dogs feel similarly. A qualitative study of 11 assistance dogs for children with autism reported several dogs resisting sleeping in the child’s bedroom—whimpering and pawing at the door—suggesting a stronger bond with a parent (the primary handler) (62). There is little evidence on whether dogs whose strongest bond is with the recipient also occasionally prefer solitude or alternative company—and how often they can act on these preferences. Encouraging positive relationships with other family members might be important, as their difficulties accepting the dog may raise relinquishment risk. For instance, partners of people with PTSD have reported struggling to adjust their caregiving role and mixed emotions about the dog (35, 47, 118, 119).

Meeting assistance dogs’ age-dependent needs and managing retirement raises further welfare concerns (36, 51, 63, 102, 117)—particularly because best practices remain unclear and much depends on whether the agency offers lifelong support beyond the assistance role. Retirement adjustment is stressful for dogs due to sudden routine changes (36, 51, 63). Discouraging or no longer rewarding assistive tasks may also elicit confusion, frustration, anxiety, and behaviour problems (51), although intermittent reinforcement during training may buffer this effect.

Retirement plans where dogs remain with the handler pose challenges including dogs’ inability to accompany the handler everywhere and adjusting to a replacement dog—potentially eliciting separation anxiety or inter-dog aggression, respectively (51). However, PTSD assistance dogs have an advantage over dogs with permanently impaired handlers—PTSD symptoms might improve enough to make a replacement dog unnecessary. Moreover, gradually reducing dog-accompanied outings and phasing out maintenance training may benefit both parties. It has been suggested that PTSD assistance dogs could hinder handlers’ habituation to trauma reminders and independent coping (11, 24, 39). Progressively fostering independence may thus facilitate the dog’s retirement transition while supporting the handler’s self-mastery during recovery. This aligns with the European standards, which recommend a progressive, phased approach to assistance dog retirement to support both the dog and handler in adjusting to the transition (78). Returning retired dogs to puppy-raisers or third-party adopters may alleviate confusion from described handler-related issues (51), but does not offset the distress of separation from their bonded person. Adapting to a new home, people, and possibly animals further amplify the stress (51).

The chronic stressors outlined—or their analogues—apply across assistance dog types (9, 25, 35, 43, 117). Specific concerns for PTSD assistance dogs include uncertainty about coping with all PTSD symptoms (9, 25) and ethical considerations when using animals in trauma response. For example, PTSD is robustly associated with dysregulated anger and elevated aggression/violence in civilian and military populations alike (1, 35, 120–122). The anger may manifest as irritability, hostility, or self-directed aggression (including recklessness, suicidality), increasing risk of interpersonal conflicts and violence (122). A review by MacManus et al. (121) found combat-related PTSD significantly associated with criminal and domestic violence among military personnel and Veterans. Notably, irritability and anger often persist post-treatment (122). Although some speculate PTSD assistance dogs may respond to violent outbursts with fear or defensive aggression (9), no studies have actually investigated how dogs cope with these symptoms or whether they may be victims of violence. The study on autism assistance dogs found dogs were often targets of child aggression and learned to distract/comfort or avoid the child depending on the distinct state of anger (62). Predicting the outcomes of their handler’s emotional states is likely a daily challenge for PTSD assistance dogs trained to calm an agitated person (35).

This highlights how assistance dogs face ambivalent situations, having to choose between trained and self-preserving behaviours. Such situations require great emotional control, as dogs are expected to remain calm and never retaliate or exhibit aggression, even under high stress (56, 62, 123, 124). For instance, similar concerns about dogs having to suppress their emotional response in challenging situations have also been raised in guide dog research (124). Research is urgently needed to assess how effectively PTSD assistance dogs maintain emotional balance while upholding training standards—and whether those bred for attentiveness to human emotions and low arousal outperform shelter-sourced dogs. Burrows et al. (62) emphasised teaching dogs that leaving the handler in potentially abusive situations is acceptable—a principle also relevant for PTSD assistance dogs. Still, as with intimate partner violence, dogs may not be in a position to escape.

Handlers’ reckless or angry behaviour not directed at the dog can also endanger them. For instance, Veterans in Williamson et al. (35) reported their PTSD assistance dog’s presence helped suppress road rage. While positive, this underscores risks for dogs paired with impulsive/reckless handlers.

Another welfare concern is that—through synchronisation, emotional contagion, or association with unpleasant human behaviour—assistance dogs may experience stress or anxiety themselves when detecting handler distress related to PTSD intrusion/hyperarousal symptoms. Several studies show dogs can detect human stress (125–128) and might respond with emotional contagion (129–132). PTSD assistance dogs are routinely trained to detect and interrupt handler’s distress episodes by comforting or distracting them. The mechanism behind alerting/disrupting tasks may even involve negative reinforcement, with dogs seeking to alleviate their own distress induced by the handler’s emotional state. These concerns are echoed in the newly published European standards, which emphasise the importance of PTSD assistance dogs’ ability to cope with high levels of handler emotional arousal while performing tasks without experiencing anxiety (64). However, specific strategies to support the dogs in these situations have not been proposed, and the impact on their emotional state remains unstudied.

Two studies on hair cortisol demonstrated that herding breeds bred for human cooperation synchronise long-term stress levels with their guardians (133), while ancient and solitary hunting breeds do not (134). Retrievers—also bred for human cooperation (134)—may similarly synchronise stress. Parr-Cortes et al. (132) found dogs exposed to human stress odors made more pessimistic choices in cognitive bias tests, indicating adverse emotional impact. Future studies should explore whether stress-synchronisation/emotional contagion presents greater welfare risks for PTSD assistance dogs of retriever breeds—and whether the selective breeding for stress tolerance sufficiently mitigates this (acknowledging individual differences may outweigh breed differences). Their simultaneous selection for sensitivity to human emotions could also factor into the equation.

Positive reinforcement-training may help PTSD assistance dogs develop positive associations with human distress (135). A meta-analysis of judgment-bias studies showed animals in favourable conditions—e.g., those trained with reward-based methods—exhibit more optimistic responses to ambiguous stimuli and greater positive affect (136). Positive associations are less likely in dogs trained with aversives or without specific training. However, in the absence of research, no data prove that even exclusively positive reinforcement-based training fully neutralises negative effects. Potential shortcomings might be leveraged by strengthening the dog’s association of human stress stimuli with reinforcers through Pavlovian conditioning, including counterconditioning (55).

Common PTSD comorbidities—like depression and substance use disorder—also pose welfare risks. Continuing a pattern in other areas we highlighted earlier, research on human-animal interactions and depression largely focuses on human benefits, with little attention to animal impact. However, some evidence indicates harm: Hunt et al. (137) found handler depression and PTSD symptoms predicted problem behaviours (e.g., excessive attention-seeking, separation anxiety) in search and rescue dogs, while Dodman et al. (138) found men with moderate depression more often practiced aversive training on companion dogs. Severe depression could also prevent the handler from offering affection, attention, or adequate care (including maintenance training, exercise/play, veterinary care). Moreover, depression and PTSD increase suicidal impulses (1, 122, 139), heightening risks of neglect, relinquishment, or abandonment. Substantial evidence shows PTSD assistance dogs significantly reduce recipient depression symptoms and suicidal ideation (6, 24, 25, 35, 43, 46, 47, 140). Nevertheless, no studies have examined whether handler depression/suicidality endangers dogs—highlighting a critical research gap.

Current understanding of dog-guardianship and substance use disorder mirrors that of depression. Research has focused on how dogs aid recovery, with preliminary evidence suggesting they provide structure, purpose, and responsibility (141). PTSD assistance dogs have also been associated with reduced substance use (35, 140, 142, 143). While these findings are promising, having an assistance dog does not necessarily preclude problematic substance use—underscoring the need for research into impacts on dog welfare.

One reassuring aspect is that many providers rigorously screen PTSD assistance dog recipients. Reported exclusion criteria include insufficient functionality to care for the dog (e.g., difficulty getting out of bed or leaving home) (47), elevated suicide/homicide risk (50, 144), history of violence/animal cruelty (42), severe substance use disorder, and certain psychiatric conditions like bipolar disorder or schizophrenia (50). Still, without standardised guidelines, screening practices vary, and optimal approaches for safeguarding dog welfare remain unstudied.

Further concerns stem from PTSD assistance dogs’ trained skills. Beyond diffusing distress and anger, interrupting nightmares may also entail long-term health risks. Burrows et al. (62) found autism assistance dogs frequently lost sleep during nights when following unsettled children, leading to exhaustion and poor performance the day after. Similar issues may affect PTSD dogs paired with handlers experiencing significant sleep perturbations and nightmares. Research is also needed on dogs’ physical safety during nightmares/flashbacks where handlers fighting imagined assailants may inadvertently hurt the dog who is trying to disrupt the episode.

Many other tasks—like “block,” “cover,” room checks, or retrieving items—seem low-risk, but observational studies are needed to confirm this. For instance, in busy areas, tasks like creating personal space or guiding a disoriented handler (who might be uncooperative) may elicit stress or pose safety risks for dogs.

PTSD assistance dog welfare can further vary depending on the tasks tailored to the handler’s needs. Mobility assistance tasks like bracing, door-opening, or wheelchair-pulling may cause discomfort, pain, and long-term musculoskeletal issues. Wheelchair-pulling particularly, is linked to serious physical concerns, including body misalignment and joint, back, and shoulder strain [see Bremhorst et al. (36), Coppinger et al. (54), Serpell et al. (63), and Coppinger et al. (92) for details]. Similarly, door-opening can strain dogs’ bodies and teeth [see Bremhorst et al. (36), Serpell et al. (63), and Coppinger et al. (92) for details]. These effects are exacerbated when the dog is overweight or wears poorly fitted/designed gear—both common among assistance dogs (36, 54, 92, 145). In guide dogs, musculoskeletal problems—often linked to suboptimal harnesses/handles—are a leading cause of early retirement (51, 102, 145). Thus, such problems not only harm dogs but can also shorten their working life, affecting the handler. As a promising development, the EU standards safeguard dogs providing mobility assistance, for example, by prohibiting their use as the primary source of wheelchair propulsion and restricting handlers from resting their weight on the dog (78).

The welfare concerns discussed above certainly do not apply to all PTSD assistance dogs. Welfare likely varies widely depending on the handler’s PTSD symptoms and comorbidities (and their severity), the dog’s background and training, their unique relationship, and the education/support provided by the agency. Some studies report handlers aware of possible shortcomings regarding meeting their dog’s needs (35) and prioritising play and rest (26). However, as no studies have systematically investigated PTSD assistance dog welfare, the severity and prevalence of the discussed welfare issues remain unknown.

### 3.2 Guiding ethical principles for ensuring the well-being of PTSD assistance dogs

Addressing the above welfare concerns in a way that minimises harm and reasonably qualifies as humane (i.e., entailing no more suffering than “necessary”) (146) may suffice for some to consider using dogs for human benefit ethically permissible. Known as the ‘welfarist’ approach in animal ethics, this view currently underpins legal regulations on animal use and treatment. In such a framing of our duties to other animals, various basic human interests enjoy priority when there is a conflict (37, 146).^6^ However, a growing perspective among ethicists recognises sentient animals as bearers of rights that should be extremely difficult to override (37, 38, 146–149).

Talk of rights ties closely to justice. Here, we lean on formal justice, which asserts that like should be regarded alike (147). If individuals are treated differently, there must be relevant dissimilarities to justify it. In the absence of such dissimilarities, relevant similarities (e.g., sentience, agency) justify equal treatment in matters where those similarities support the relevant protections, rights, or moral regard (147). Importantly, talk of relevant similarities and dissimilarities does not track different species identities *unless* what makes these species distinct are morally relevant. In biology, species categories are not designed to do moral work, as, on the whole, they merely pick out individuals with to-be-specified genetic similarities indicative of individuals who can successfully procreate (147). To be a possessor of rights, then, does not require that the rights bearer is human. Indeed, if it did, rights theory would have to abandon the conceit of being universal, but that comes at too high a price as it excludes *any* non-humans, *regardless* of capacities or interests, from the community of rights bearers. As mentioned in an early footnote, rights are, as we understand them and as they tend to be understood in rights theory, best explained as protections of basic interests or agential capacities (37, 38, 150). Where other animals possess relevantly similar interests or agential capacities to currently protected humans, formal justice and a commitment to logical consistency requires extending relevantly similar rights to the relevant animals. Under this scope of “rights talk,” domestic dogs clearly qualify as rights bearers.

This rights-based orientation dissents from Utilitarian, or broader consequentialist, arguments that justify animal exploitation if it substantially enhances human well-being and promotes greater overall societal good (37, 38, 149). Such consequentialist reasoning appears in PTSD assistance dog literature—for instance, Krause-Parello et al. (25) argue that the many people benefiting from these dogs and varying attitudes toward animal welfare justify proceeding with their use despite dogs’ unconfirmed safety and capability to effectively cope with all PTSD symptoms.

To add further clarity, our thinking here is heavily informed by both a rejection of anthropocentric speciesism and a commitment to a just distribution of rights (i.e., aligning with formal justice), where rights are currently, and incontestably, ascribed to humans between birth and death. Anthropocentric speciesism picks out moral frameworks that prioritize human interests over the interests of other animals for no other reason than the relevant individuals are humans (31, 151). As we have already indicated, such speciesism ignores relevantly similar animals—where they are relevantly similar—and so violates the formal justice commitment (and a commitment to logical consistency). Being sentient, PTSD assistance dogs obviously possess rights due to having basic interests (including the interest to thrive; see introduction) relevantly similar to already protected humans. Upholding these rights is the duty of assistance dog training organisations and handlers. Thus, PTSD assistance dog well-being—having a good life—is distinct from welfare and equally ethically significant. In light of our stated framework, we hold that both welfarist and Utilitarian (or broadly consequentialist) approaches are ethically inadequate and that a lack of evidence of harm, mere harm minimization, and the supposed greater good achieved through human benefit do not justify exploiting PTSD assistance dogs (31, 149, 152).

The next subsection explores long-term ethical values to ensure interspecies justice for PTSD assistance dogs (146), aiming to create work environments that not only minimise risks but are also *good* in that they contribute to these dogs’ well-being and enable them to flourish (153). These principles guide us toward more ethical (non-exploitative) human-animal relationships, respecting that assistance dogs, like other animals, have lives of their own beyond serving humans (37, 38, 152).

#### 3.2.1 Respect for PTSD assistance dogs’ agency and dissent

An important advance in animal well-being is the shift toward respecting animal agency (31, 84, 146, 154). All sentient beings are agents with desires/preferences and the capacity to make choices based on those desires/preferences (146, 153). Work conditions promoting well-being should offer animals opportunities to exercise agency by having some control over their circumstances (31, 84, 146, 152, 153). This could be achieved by allowing animals to make choices, provided these choices are not self-destructive (31, 83, 84, 148, 153, 155).

A myriad of options exist to give PTSD assistance dogs opportunities to exercise choice in their daily lives. Providing options among essential resources, like treats, toys, or bedding supports well-being even within limited selection (83). During down-time, dogs should be free to choose whose company or which room to stay in, whether to be indoors or outdoors, or seek privacy. Research shows animals considerably benefit from having such choices even if not utilising them (148). Down-time should also offer ample opportunities for species-specific behaviours (83), like running free, exploring surroundings, or interacting with other animals (31, 38). Walks can support agency if dogs can choose directions and freely engage in sniffing when feasible/safe. It is encouraging that the EU standards recognise the latter need by mandating daily leisure walks and a minimum of weekly off-leash free-running sessions, during which sniffing, playing, and interacting with other dogs is encouraged, and by requiring sufficient daily mental and physical rest (64, 78).

Ensuring sufficient leisure time for PTSD assistance dogs considerably enhances their well-being by offering more control over their lives (31, 83, 148). However, respecting animal agency should also extend to their work. Some ethicists argue animals should choose their work (148, 153)^7^. Assistance dog screening partly allows this by selecting dogs’ career paths based on their individual traits, personality, and skills, excluding those reluctant or unsuited to the tasks and interactions (153, 155) (WCC, personal communication, April 25, 2024).

Cochrane (153) further argued that good work allows animals some control over their work tasks. The array of PTSD assistance dog tasks is diverse enough to enable dogs to choose which they are willing to perform. Many agencies likely consider a dog’s enthusiasm for specific tasks when assembling their repertoire. However, since tasks are tailored to the handler’s needs, the dog’s input is unclear. Ideally, the dog’s preferences should match the recipient’s needs.

Another important step in respecting PTSD assistance dogs’ agency is institutionalising a dissent model (148, 154). In human bioethics, the gold standard is informed consent—which requires being as fully informed as possible (relative to development and level of understanding) of all the benefits/risks associated with the intervention and a freedom to withdraw at any time (148). When individuals are incapable of providing informed consent, assent—willing participation with sufficient understanding—is acceptable if accompanied by a legal guardian’s (or other legally appropriate third party’s) informed consent (148, 154). For very young children and people with significant cognitive impairments, sustained dissent—unwillingness to participate, expressed by repeated attempts to remove themselves from the situation—is increasingly favoured (148, 151). Unlike consent or assent, sustained dissent does not require understanding the intervention’s goals, risks or benefits; expressing a sustained unwillingness to experience distress, pain, or suffering is enough to warrant exclusion from the relevant activity (148, 154). This helps prevent harm from overriding an individual’s expressed preferences given the aversiveness of what they are (expecting) to experience (151).

Some scholars seek to adapt these human bioethics concepts for animal bioethics. Given uncertainty about animals’ ability to provide informed consent or meet the full requirements of assent, several scholars propose sustained dissent as the gold standard in animal bioethics (148, 151, 155). Others, like Jones (156), contend that the current human-centric definition of consent is inapplicable to animals like dogs but advocate for a species-specific definition. Jones (156) defines consent as, “the voluntary agreement for something to occur [while being connected to agency] in that it may afford dogs the freedom to choose when and how to interact and when to opt out” (p. 10). She notes that, through learning, dogs can make informed choices in some contexts and effectively communicate their willingness to participate (e.g., cooperative care); other contexts—like medical interventions—may require guardian (or other appropriate third party) decisions based on the dog’s best interest, aligning with assent (156). Similarly, Blattner (148) proposes an embodied consent model which she thinks resembles informed consent and suggests that animals’ consent to participate could be inferred by interpreting their communication (including body language and vocalisations)—although she acknowledges animals may not fully grasp the risks and benefits of the activity. Blattner (148) therefore recommends prioritising respect for dissent, while working toward broader consent and assent models.

To avoid misaligning the terms, so that the constraints on how these terms are used in human bioethics are not sufficiently captured in animal bioethics, this paper focuses on sustained dissent as a starting point in working with assistance dogs. Until more is known about dogs’ capacity for consent or assent, respecting sustained dissent is appropriately responsive to dogs’ expressed preferences and reflects a commitment to their well-being. Dogs can express sustained dissent in various ways (148, 153, 155). They regularly do so by disobeying commands, decelerating work, exhibiting avoidance behaviours, taking unauthorised breaks, objecting vocally, damaging equipment, or refusing to work under inadequate external (e.g., heat) or internal (e.g., illness) conditions (148).

Work environments aligned with interspecies justice should allow PTSD assistance dogs to stop or refuse work by honouring their sustained dissent (148), similar to how human workers can quit or refuse tasks. ADI-accredited providers implement this in part through annual recertification. However, it remains unclear whether yearly interval is optimal, and how effectively the varying follow-up care of different agencies compensates for this. The newly published EU standards also emphasise respecting sustained dissent by recommending the withdrawal of dogs exhibiting persistent stress or anxiety during training/work; however, they do not outline how this principle should be implemented (64, 78). Ng and Fine (51) recommend biannual assessments, particularly for older dogs. Some non-accredited organisations, however, lack regular recertifications altogether or conduct them infrequently, like every three years.

Blattner (148) and Coulter (155) argue that working animals should have the right to dissent at any time, enabling them to promptly cease work that causes discomfort or reluctance*—*just as human sustained dissent should be respected. Establishing respect for sustained dissent as a standard work condition for PTSD assistance dogs requires knowledge of and responsiveness to canine communication (148, 155). While recipients are normally educated on canine care/communication during pair-training, it is unknown how often dogs’ daily expressions of dissent—like stress signals or avoidance behaviours—are acknowledged. Burrows et al. (62) reported that one autism assistance dog’s reluctance to wear his service jacket was dismissed as laziness, though the jacket was later found to have become too small, causing discomfort. Similar reports of mobility assistance dogs in obvious distress while pulling wheelchairs or opening heavy doors (54), cast doubt on how well assistance dogs’ dissent is respected in practice.

#### 3.2.2 Fostering enjoyable and beneficial work for PTSD assistance dogs

Additional safeguards against assistance dog exploitation involve promoting the dogs’ well-being by ensuring they enjoy and benefit from their work (153, 155). Like good human jobs, good work for animals should minimise risks, foster pleasure, and offer meaning (146, 153). Cochrane (153) suggests that pleasurable animal work requires sufficient task variability to engage a range of enjoyable skills. Unlike, say, scent-detection or sled dogs focused on one task, PTSD assistance dogs use a broad skill set: olfactory and visual acuity to detect intrusion/hyperarousal symptoms (125), social skills to calm their handler and facilitate social interactions, fetching objects/medication, cognitive skills for tasks like “block”/“cover” or dialling emergency contacts, and, sometimes, physical abilities for mobility support.

However, a distinct question is whether these tasks are enjoyable or meaningful for the dogs. This matters both for their well-being and assistance effectiveness, as enjoyment affects motivation. Reflecting this, the European standards emphasise that training records shall document whether dogs enjoy particular assistive tasks and display natural aptitude for them (64). While screening potentially selects dogs who enjoy these tasks, many assistance tasks fall outside canine species-specific behavioural repertoire, offering little internal motivation (55, 92). Unlike sled or sheep dogs driven by innate motor patterns that make performance inherently rewarding (54, 63, 92), assistance tasks often lack relevance or intrinsic benefit to dogs (e.g., switching on lights, retrieving medication). Some, like wheelchair-pulling or door-opening, may even induce physical distress (54, 63, 92).

Due to the lack of internal motivation, biological relevance, and explicit reward, the motivation to perform such tasks must be generated externally (55, 92), relying heavily on operant conditioning. This parallels human labour, where difficult or unpleasant tasks are driven by external incentives (e.g., salary, academic degrees). As noted, aversive techniques are often used to “motivate” dogs, especially for ongoing (i.e., without clear beginning/ending) or physically uncomfortable tasks. However, aversive training is unequivocally unethical, contradicting principles of animal agency, respect for sustained dissent, and enjoyable work. Although even continuous positive reinforcement might not prevent a trained skill from diminishing if it diverges from intrinsic behaviours (63, 92), positive reinforcement-based training is more likely to increase motivation and make work more enjoyable.

Positive reinforcement-based training also aligns with other ethical principles of providing animals good work. Based on voluntary cooperation, it lets the animal choose whether to perform the task and receive the reward, or not perform and forgo it (90, 99, 100, 154, 156). Yet even positive reinforcement can sometimes become coercive (151), especially under high external pressure (such as the high demand and low supply of PTSD assistance dogs), where the principle of voluntary cooperation is overlooked and animals are pushed to work despite frustration or diminished motivation. In such circumstances, institutionalising a work ethic that respects sustained dissent is crucial. Furthermore, positive reinforcement-training can address the primary weakness of the dissent model—while dissent allows refusal, it does not confirm willingness (148). Truly voluntary, non-coercive, positively reinforced work can reflect assent (or acquiescence), as the animal chooses to work for a reward, indicating some motivation (83).

One non-coercive approach to boost dogs’ motivation for assistive tasks is to explore whether selectively bred dogs are more intrinsically motivated for PTSD assistance work, as proposed by WCC (60). Other strategies include broadening breed selection (including cross-breeding) to identify dogs with greater motivation for specific tasks or, as Gadbois and Reeve (157) suggest, considering breed differences in dopaminergic systems to select more work-motivated dogs. If no efforts succeed to make these tasks enjoyable and non-coercive methods fail, using dogs for such purposes should cease. That dogs *can* be trained for these tasks does not mean that they *should* be—especially if it presents a morally unacceptable risk of harm. For example, motorised/electric wheelchairs can eliminate the need for dogs to pull them (78). While occasional frustration and discomfort are natural parts of learning—much like in human skill acquisition—PTSD assistance dog training and work must be enjoyable overall to be ethically acceptable (153, 155). Tasks that remain consistently unpleasant and distressing even in fully trained dogs should be excluded.

Another way to promote benefits for PTSD assistance dogs involves deliberately nurturing their bond with the recipient. An increasing body of research shows the human-dog bond offers substantial advantages for people with PTSD (9, 24–26, 29, 47). Strong recipient-dog bond also improves assistance dog training outcomes, work performance, and the overall success of the partnership (26, 29, 35, 47, 55). Accordingly, most PTSD assistance dog agencies already prioritise this bond, dedicating varying amounts of time to its development (26, 35, 47, 50).

A strong recipient-dog bond should also guarantee benefits for the dog, as a human-animal bond is, by definition, a mutually beneficial positive relationship that promotes both partners’ well-being and health (35, 55, 158). However, few studies have investigated how bond quality affects assistance dogs and whether such bonds are consistently present. Lane et al. (80) found no conclusive evidence that mobility assistance dogs’ welfare was influenced by their relationship with recipients. A review by Hall et al. (55), however, reported that human-dog bonds based on secure attachments are linked with dogs’ lower stress and neuroticism, and greater persistence in novel contexts. It also remains unclear whether instrumentalising dogs is detrimental to their well-being in contexts where bonding is encouraged. Overall, more research is urgently needed on how attachment styles and recipient-dog relationship dynamics can advance ethical, sustainable assistance dog practices (55).

Finally, Coulter (155) argues that enjoyable work includes supporting the animal’s well-being outside working hours—both daily and across their lifetime. While we have addressed daily needs like quality downtime, ethical work standards require a holistic approach that also prioritises well-being before and after their formal careers. Coulter (155) illustrates this by pointing to human parallels like childcare, education, pensions, and elder care. If the welfare issues outlined in subsection 3.1 are adequately mitigated, the upbringing (by dedicated foster families) and training of PTSD assistance dogs can significantly enhance their lifelong well-being by preparing them for future roles. This rationale implies that dogs entering the program as puppies may enjoy better overall well-being throughout their working lives than those recruited later.

Additionally, much can be done to promote an enjoyable retirement for former assistance dogs, regardless of living arrangements. Alongside facilitating retirement adjustment, providing ample enrichment, stimulation, and activities that match their interests is essential (51). This priority is acknowledged in the new European standards, which require a dedicated health and well-being plan for retired assistance dogs (78). A major unresolved question—reflected in the lack of standards—is when to retire an assistance dog. While illness impairing work (e.g., musculoskeletal disease, cognitive dysfunction) clearly warrants retirement (51, 102), truly enjoyable retirement requires retiring dogs while still healthy and free of debilitating conditions. This implies enforcing retirement before signs of illness appear (51), creating a potential conflict of interest, as retiring a fully functional dog hardly aligns with the recipient’s interests.

To fulfil our ethical duty to provide good retirement, data on the age at which assistance dogs retire and the reasons for retirement should be systematically collected and analysed. Such information is essential for understanding how best to support healthy ageing and to determine the optimal retirement age for these dogs (102). Ng and Fine (51) recommend retiring dogs during the final 25% of their life expectancy—typically their senior life stage. This accounts for breed-specific lifespans and roughly compares to human retirement. In addition to monitoring behavioural changes or performance decline, they suggest measuring hair cortisol to detect rising overall stress and conducting surveys to recognize declines in quality of life (51). These tools could help determine when to retire otherwise healthy dogs.

### 3.3 Conclusive suggestions for improving the welfare and well-being of PTSD assistance dogs

To conclude, it is important not only to recognise the potential welfare risks inherent in the training, placement, and working lives of PTSD assistance dogs, but also to outline concrete measures that can promote their well-being. The recommendations outlined in Table 1 provide a framework for agencies, trainers, recipients, and policymakers to adopt. They are divided into two categories: welfare (understood as the protection of dogs from harm and suffering) and well-being (understood as the active promotion of positive states and quality of life). Implementing these measures can help ensure that assistance dogs not only avoid harm but also thrive, thereby strengthening the sustainability and ethical legitimacy of PTSD assistance dog programs.

**Table 1:** Recommendations for safeguarding and promoting the welfare and well-being of PTSD assistance dogs.

Domain	Recommendation
Welfare: Ensuring the protection of physical, psychological, and emotional welfare	
Equal attention to psychological and emotional welfare	Agencies and handlers should prioritise dogs’ psychological and emotional welfare on par with their physical health.
Veterinary care and monitoring	Agencies should organise regular veterinary inspections, recertifications, and lifetime follow-up care of assistance dogs.
Legal safeguards	Agencies should retain (at least partial) legal control to intervene and reclaim dogs whose welfare is compromised.
Stable living arrangements	Minimise social and environmental disruptions caused by separations from or changes of the dog’s family/home.
Kennel management	Limit time spent in agency kennels; ensure enriched, low-stress kennel environments that provide adequate stimulation.
Training methods	Prohibit dominance-based, aversive, or coercive methods.
Task selection	Omit tasks that remain consistently unpleasant, aversive, or harmful to dogs (e.g., some mobility assistance tasks).
Monitoring recipient-dog dynamics	Implement rigorous follow-up routines to assess how dogs are affected by recipients’ PTSD symptoms and comorbidities.
Recipient education	Enhance recipients’ skills in dog training/handling, and understanding canine behaviour, communication, and welfare.
Reject instrumental views	Avoid framing assistance dogs as mere tools.
Well-being: Promoting positive states and quality of life beyond minimising harm	
Treating dogs as agents	Create conditions that allow dogs to exercise choice both during work and leisure.
Respecting sustained dissent	Institutionalise respect for dogs’ sustained dissent—both during recertifications and daily work routines.
Quality down-time	Ensure daily opportunities for rest, species-specific behaviour, pursuing individual interests, and control over social/physical environment.
Enjoyable work	Ensure that assistance tasks and the training process are enjoyable for dogs; prioritise nurturing the recipient-dog bond.
Proper preparation	Provide prospective assistance dogs with an upbringing involving thorough socialisation and ample experiences that foster resilience and coping with novelty.
Retirement with dignity	Plan for enjoyable retirement through gradual transition, health free from debilitating conditions, and daily enrichment matched to the dog’s needs and interests.

## 4 Conclusion

This paper highlights a major research gap in PTSD assistance dog literature: while existing studies focus on benefits to people, to the best of our knowledge, no meaningful attention has been given to how this intervention affects the dogs. Using an ethics-oriented approach, we considered whether PTSD assistance dog interventions promote the well-being of both partners or risk exploiting animals for human gain.

The lack of standardisation and consensus on best practices for PTSD assistance dog selection, rearing, training, and follow-up raises significant animal welfare concerns. These dogs face many of the same welfare risks as other assistance dogs: constant work, social and behavioural deprivation, lack of routine and control over their life, instability of social and physical environment, disruption of close relationships, harsh training, and potential distress from certain tasks. Some PTSD symptoms and comorbidities might further endanger or challenge the dogs.

Applying an animal-rights framework, we proposed long-term ethical constraints to provide PTSD assistance dogs with work conditions that minimise risks of harm, uphold interspecies justice, and contribute to their well-being, enabling a good life. These include better recognising that dogs are agents, respecting their sustained dissent, offering ample rest and opportunities to pursue individual and species-specific interests, and ensuring enjoyable work that benefits the dogs themselves. The latter requires a strong recipient-dog bond, force-free training/handling, tasks the dog finds pleasant, and a healthy, fulfilling retirement.

Overall, available information indicates wide variation in PTSD assistance dogs’ well-being, influenced by each dog’s background, training/handling methods, provider follow-up, the recipient’s symptoms and comorbidities, the dog’s tasks, the recipient-dog relationship, and adherence to the ethical principles mentioned. Nevertheless, this paper remains theoretical, flagging prospective welfare and ethical issues for future study. Empirical research is urgently needed to evaluate PTSD assistance dog interventions from the dog’s perspective, gauge these concerns, and establish best practices to enhance their working conditions.

The development of nationally recognised assistance dog standards—at least in the EU and Australia—is encouraging, as it signals growing attention to dog welfare and may stimulate much-needed research (e.g., on agencies’ adherence to these standards). However, in addition to standards and regulations, achieving the improvements outlined in this paper also depends on broader societal commitments: valuing and supporting people with PTSD (and other mental health conditions) and ensuring adequate funding for assistance dog programmes. Combined, these efforts are crucial for advancing PTSD assistance dog interventions toward more just partnerships that prioritise and protect the well-being of both partners.

## Footnotes

1 Our talk of “most humans” (instead of “all humans”) may cause some confusion to non-specialists unfamiliar with rights theory. In this paper, when we talk about “humans” we are referring to members of our species, *Homo sapiens*. This means that human zygotes and embryos are properly referred to as “humans” but they are not obviously bearers of rights, be they understood as moral or legal rights. Rights, as we understand them here [and this is something of a traditional stance in rights theory (150)], are best explained as protections of either basic interests or agency (37, 38), and so any human lacking interests or agential capacities lacks rights. It must be stated for clarity that, fundamentally, talk of basic interests refers to that which constitutes welfare—such that satisfying interests constitutes positive welfare while violating them constitutes negative welfare (151)—and so is compatible with disabled humans, and even late-term human fetuses, possessing interests and therefore rights.

2 We acknowledge that, while establishing standards is important to integrate canine-assisted interventions into PTSD treatment protocols and control for factors like dog welfare, standards should also remain flexible enough to allow for the ongoing development of best practices. This concession does not substantially affect what we will later discuss.

3 During pair-training, the recipient and the dog practice working together as a team. Pair-training usually involves handler training and facilitating recipient-dog bonding, but it can also include further customised training based on the recipient’s specific needs (35, 42, 47) (WCC, personal communication, April 25, 2024).

4 Originally developed by the British Farm Animal Welfare Advisory Committee in 1965, the Five Freedoms include “Freedom from Hunger and Thirst”; “Freedom from Discomfort”; “Freedom from Pain, Injury, or Disease”; “Freedom to Express Normal Behaviour”; and “Freedom from Fear and Distress” (159).

5 The domains in the Five Domains Model are “Nutrition,” “Physical Environment,” “Health,” “Behavioural Interactions,” and “Mental State” (86, p. 1).

6 Our talk of a “welfarist” ethics should not be confused with our talk of animal welfare science. *Any* ethics orientation that takes other animals’ interests seriously will attend to what, about these animals, can be known through the relevant animal welfare sciences.

7 What we mean by advocating for dogs to choose their work is that they should voluntarily engage in all the activities involved in their career path. This does not require dogs to understand the goals of their service. What matters is that they enjoy performing these activities and spending time in the company of the individuals they work with, and that each dog’s role matches what they are best suited for regarding their individual interests and preferences.
